# Cancer‐associated fibroblasts mediate resistance to neoadjuvant therapy in breast cancer

**DOI:** 10.1002/ctm2.1779

**Published:** 2024-07-20

**Authors:** Zihan Xia, Stephanie Vermeulen, Ujjwal Suwal, Pekka Rappu, Jyrki Heino, Felix De Vuyst, Sandor Dedeyne, An Hendrix, Hannelore Denys, Olivier De Wever

**Affiliations:** ^1^ Department of Human Structure and Repair Laboratory of Experimental Cancer Research, Ghent University Ghent Belgium; ^2^ Cancer Research Institute Ghent Ghent Belgium; ^3^ Department of Life Technologies University of Turku Turku Finland; ^4^ Department of Medical Oncology Ghent University Hospital Ghent Belgium

Dear Editor,

We conducted a comprehensive bioinformatic analysis combined with empirical experimental evaluation, highlighting the critical role of cancer‐associated fibroblasts (CAFs) in the poor outcomes of breast cancer (BC) patients undergoing neoadjuvant chemotherapy (NAC).

BC stands as the most commonly diagnosed cancer among women worldwide.^1^ NAC is the standard treatment for locally advanced BC,^2^ and response to NAC is associated with patient prognosis.^3^, ^4^ Despite similar tumour histology, stages, and uniform NAC, treatment response among BC patients significantly varies. While numerous molecular biomarkers have been investigated to predict NAC response,^5^ these do not consider that cancer cells reside within a complex tumour microenvironment (TME) composed of non‐malignant cells that mediate cancer progression and NAC resistance.^6^, ^7^ Given the complex interplay between stromal cells and cancer cells, we identified, explored and functionally validated stromal cell‐derived molecular signatures as indicators of NAC response (Figure 1A).

**FIGURE 1:**
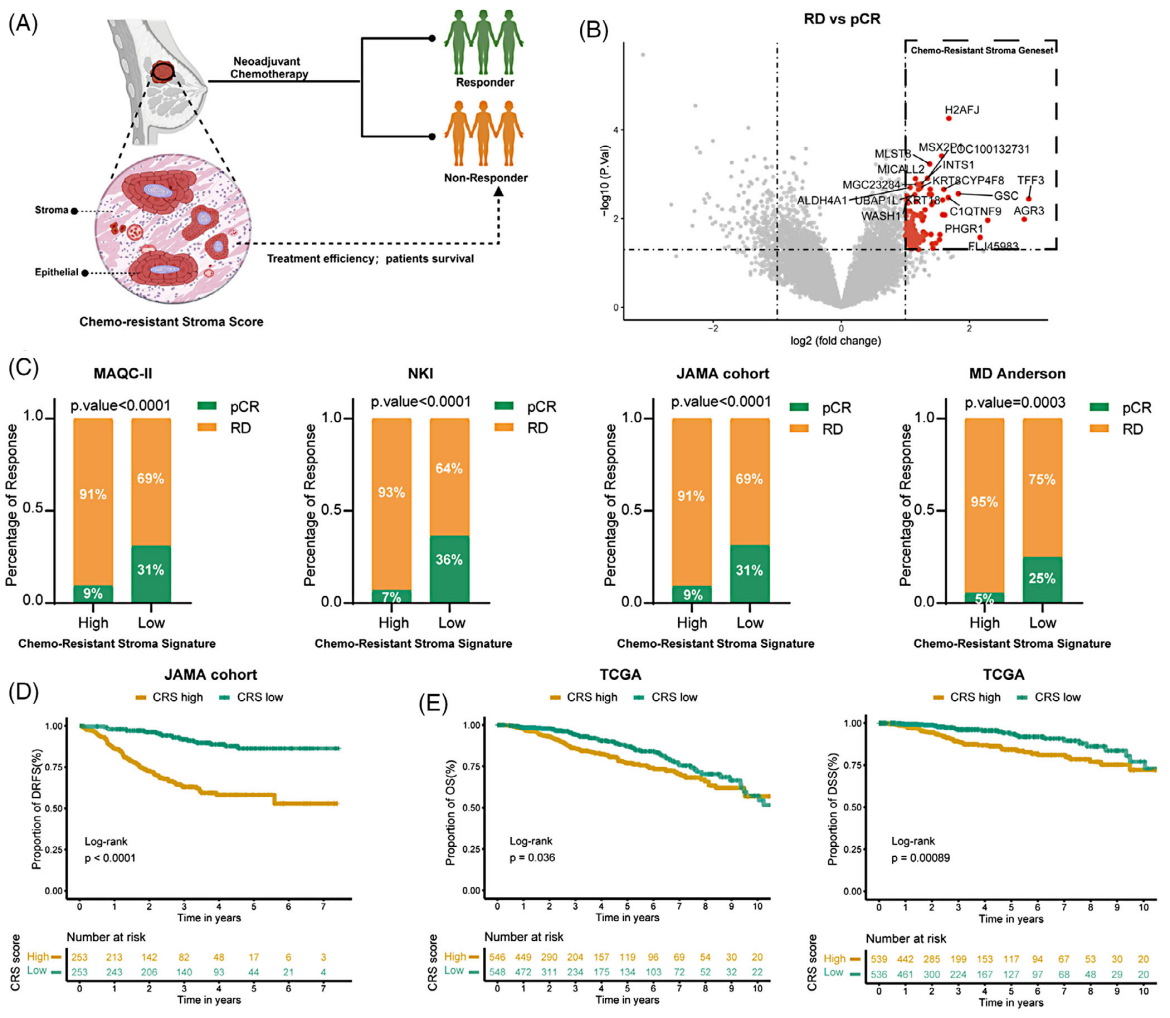
Chemo‐resistant stroma indicates clinical outcome in breast cancer patients. (A) This illustrative figure highlights that the chemo‐resistant stroma (CRS) signature can serve as an indicator of clinical outcome in breast cancer patients receiving neoadjuvant chemotherapy (NAC). (B) The volcano plot illustrates significantly differentially expressed genes in laser capture micro‐dissected stromal cells from residual disease (RD) patients (*N* = 35) compared with those from pathological complete response (pCR) patients (*N* = 9) in pre‐therapeutic tumour biopsies (GSE143846 dataset). (C) The NAC response rate was analyzed in four datasets and categorized based on high and low CRS signatures. The CRS enrichment scores were calculated using the single‐sample gene set enrichment analysis (ssGSEA) algorithm and the patient's stratification was based on the median value. Statistical analysis was conducted using Fisher's exact test. (D) Kaplan‐Meier plots depict the proportion of distant relapse‐free survival (DRFS) in patients from the JAMA cohort classified according to CRS risk score based on the median value. (E) Kaplan‐Meier plots demonstrate the proportion of overall survival (OS) (left) and disease‐specific survival (DSS) (right) in patients from the TCGA‐BRCA cohort classified according to CRS risk score based on the median value. Statistical significance for differences in the Kaplan‐Meier plots between the two groups was determined using the log‐rank test.

First, a chemo‐resistant stroma (CRS) signature was identified as a list of differentially higher expressed genes in laser capture micro‐dissected stromal cells from pre‐NAC biopsies derived from BC patients with residual disease (RD) compared to patients with pathological complete response (pCR) (Figure 1B). BC patients from four cohorts (Table S1) were stratified into high and low CRS signature groups. For all cohorts, RD patients were associated with high CRS signatures (Figure 1C). Next, we screened the CRS gene signature using the Lasso penalized Cox regression analysis and delineated an 11‐gene‐based CRS risk model (Figure S1A). Kaplan‐Meier analysis revealed that BC patients demonstrating a low CRS risk score exhibited a significantly higher distant relapse‐free survival (DRFS) rate (Figure 1D). The CRS risk score was identified as an independent risk factor through multivariate Cox regression analysis (Figure S1B) in the JAMA cohort. These findings were subsequently validated in the TCGA‐BRCA cohort (Figure 1E and Figure S1C). Taken together, these results demonstrate the robustness of the CRS signature in indicating BC patient survival and response to NAC.

To further confirm the role of the stromal part in NAC response, we performed a comprehensive transcriptomic analysis of pre‐treatment biopsies from BC patients in the JAMA cohort (Figure 2A and Figure S2A). However, principal component analysis did not differentiate pCR or RD patients (Figure 2B), as cancer cells are the main components within the tumour biopsies further argues the possibility that stromal cells rather than the cancer cells determine NAC response. Indeed, an increased stroma score, but not an immune score, and elevated epithelial‐mesenchymal transition (EMT), extracellular matrix (ECM), collagen fibril organization and CAF (the main source of ECM^8^) signatures were identified in pre‐treatment biopsies from RD patients (Figure 2C,D and Figure S2B). In contrast, there was no enrichment of signatures associated with normal fibroblasts, endothelial cells or T cells in RD patients at baseline (Figure S2C), further highlighting the relevance of CAFs in impaired NAC response. In JAMA and TCGA‐BRCA cohorts, BC patients exhibiting a high CAF score were significantly correlated with an increased enrichment of the EMT signature (Figure 2E). EMT is a prominent process that plays a significant role in cancer cell plasticity and NAC resistance.^9^ Kaplan–Meier analysis revealed that BC patients with a high EMT score experienced shorter DRFS periods (Figure 2F). In addition, a strong positive correlation was observed between EMT signature and signatures for stroma, CAF, ECM as well as collagen formation (Figure 2G and Figure S2D). Collectively, these data strongly support the notion that within the stromal part particularly CAFs confer NAC resistance.

**FIGURE 2:**
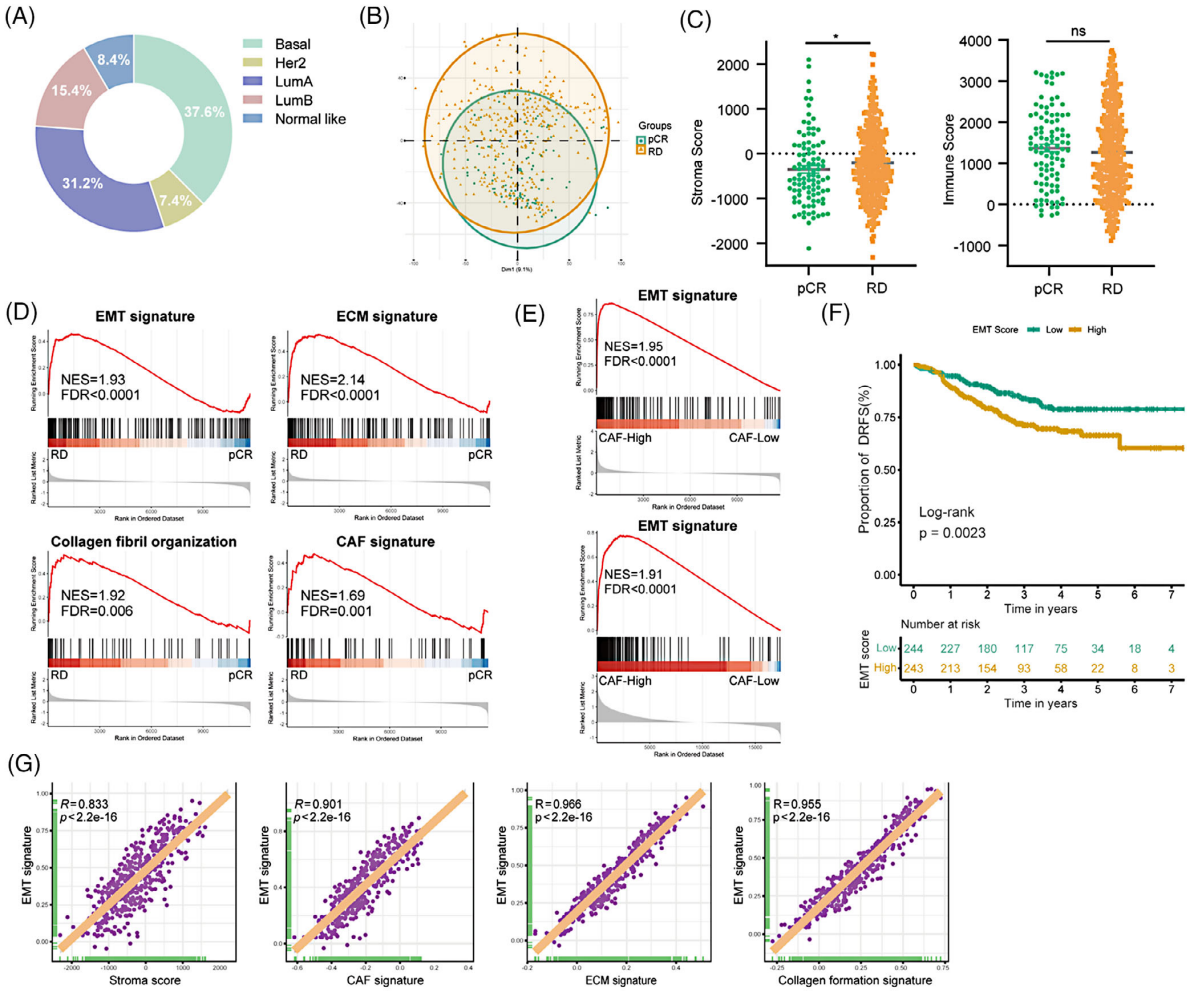
Stromal cells, particularly cancer‐associated fibroblasts (CAFs), confer neoadjuvant chemotherapy (NAC) resistance. (A) The pie chart displays the PAM50 classification in 487 breast cancer patients treated with NAC (JAMA cohort). (B) The principal component analysis (PCA) plot represents the global transcriptomic analysis of pre‐treatment tumour biopsies from the JAMA cohort, categorized into pathological complete response (pCR) (*N* = 99) and residual disease (RD) group (*N* = 388). (C) Comparison of stroma and immune scores between the pCR and RD groups using the ESTIMATE algorithm. Statistical significance was determined using the Wilcoxon test, with * indicating a *p*‐value ≤ .05. (D) Gene set enrichment analysis (GSEA) for epithelial‐mesenchymal transition (EMT), extracellular matrix (ECM), collagen formation and cancer‐associated fibroblast (CAF) signatures from the JAMA cohort. (E) GSEA enrichment plots represent the hallmark EMT gene set, comparing breast cancer patients with high versus low CAF enrichment scores in JAMA (upper panel) and TCGA (lower panel) cohorts. (F) Kaplan‐Meier plots depicting the proportion of distant relapse‐free survival (DRFS) in patients with high EMT enrichment scores versus low scores. EMT enrichment scores were calculated using the ssGSEA algorithm and differences between the two groups were assessed using the log‐rank test. (G) Correlation analysis of scores for hallmarks EMT signatures versus scores for stroma, CAF, ECM, and collagen organization signature in JAMA datasets. Purple plotting symbols represent individual samples. Correlation coefficients (R) and *p*‐values were calculated using Spearman's correlation analysis. Significant enrichments were determined based on a normalized enrichment score (NES) threshold of > 1.5 and a false discovery rate (FDR) < 0.05.

To elucidate the underlying mechanisms through which NAC modulates stromal functions and to assess the impact of these processes on cancer progression, we compared transcriptomic data of 20 paired pre‐ and post‐chemo BC biopsies. GSEA indicated that NAC induced signatures of EMT, ECM, collagen formation as well as CAF (Figure 3A and Figure S3A–C). Further analysis also revealed a higher stroma score and higher CAF abundance in post‐chemo BC samples. In contrast, NAC treatment had no effect on the infiltration of T cells, macrophages and endothelial cells (Figure 3B and Figure S3D). Paclitaxel (PTX), a microtubule‐stabilizing agent causing mitotic arrest, is a widely used component of NAC in BC patients. We next assessed PTX cytotoxicity against BC patient‐derived CAFs and BC cell lines cultured under three‐dimensional spheroid conditions. Corroborating with bioinformatic analysis, we observed that CAFs were highly resistant to PTX in contrast to cancer cells (Figure 3C). In line with microtubule activity in migratory and contractile CAF, we observed a drastic inhibition of contractility, migration, outgrowth, and invasion but not metabolic activity (ATP content) and cell death (Figure 3D and Figure S3E–I).

**FIGURE 3:**
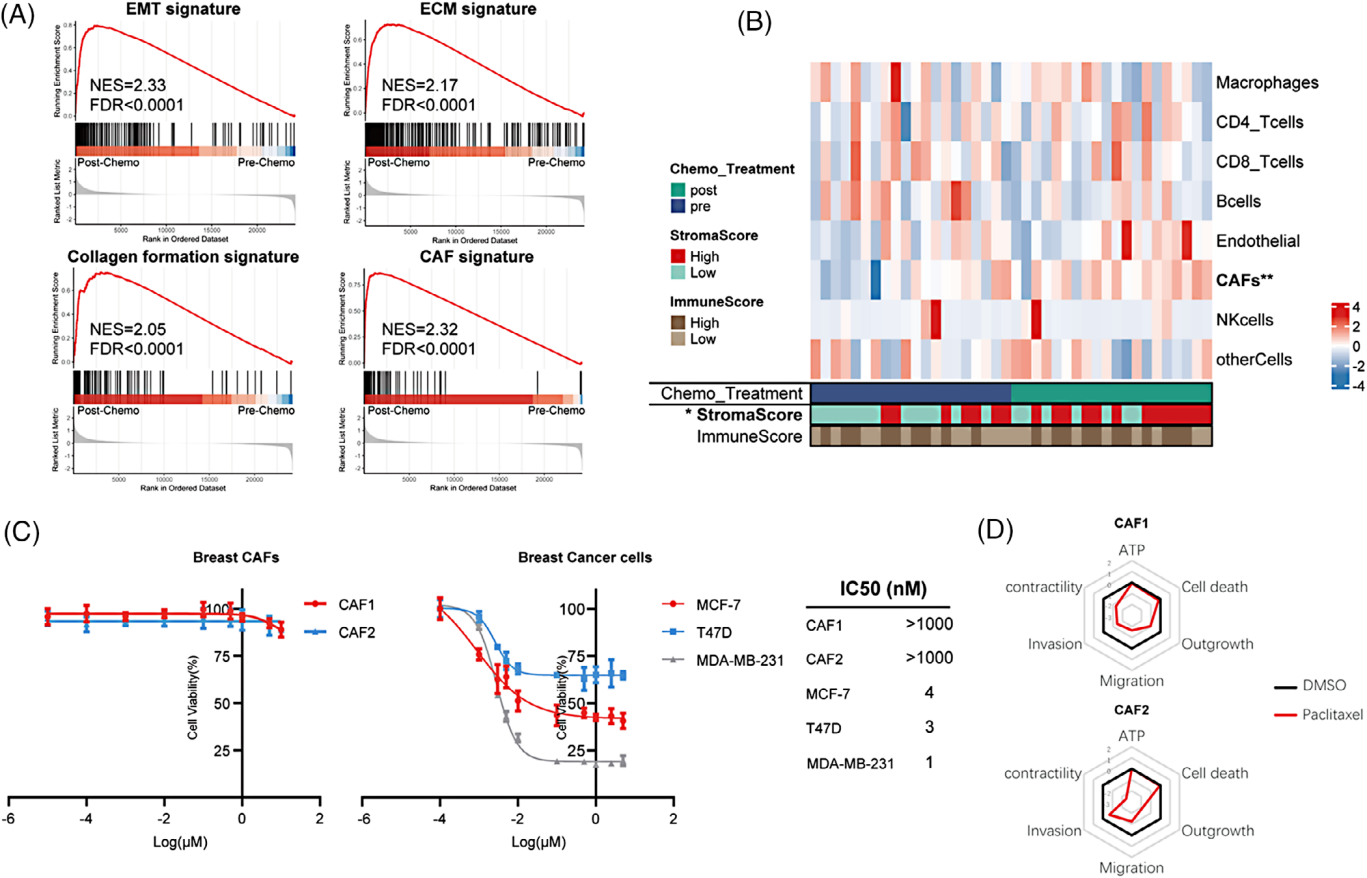
Cancer‐associated fibroblasts (CAFs) persist during neoadjuvant chemotherapy (NAC). (A) Gene set enrichment analysis (GSEA) for epithelial‐mesenchymal transition (EMT), extracellular matrix (ECM), collagen formation and CAF signatures using GSE191127 datasets: paired pre‐chemo (*N* = 20) and post‐chemo (*N* = 20) samples. Significant enrichments were determined based on a normalized enrichment score (NES) threshold of > 1.5 and a false discovery rate (FDR) < 0.05. (B) Heatmap of CAF, immune and endothelial cell infiltration in 20 paired post‐ versus pre‐chemo breast cancer biopsies based on EPIC, and the immune and stromal scores based on the ESTIMATE algorithm. Differences in cell fractions were assessed using the Wilcoxon test. (*p‐value ≤ .05; **p‐value ≤ .01). (C) Two breast CAFs and three breast cancer cell lines were subjected to treatment with paclitaxel (PTX) at various concentrations for 48 h. The IC_50_ values for each cell type were determined. (D) Spider plots illustrate the metrics of six functional phenotypes in two breast CAFs with or without PTX (10 nM) treatment. A lower Z‐score indicates a lower metric value.

Proteomic analysis of CAF spheroids revealed a reduced presence of cell cycle‐associated proteins (Figure 4A) as well as an induction of senescence signature in PTX‐treated CAFs (Figure 4B). In accordance, senescence induction was also identified in post‐chemo tumour biopsies (Figure 4C). Flow cytometry demonstrated a G2/M arrest (Figure 4D) and immunoblot analysis revealed higher protein levels of senescence markers p16, p21 and cyclin D1 in CAFs following PTX treatment (Figure 4E). Increased β‐galactosidase activity further confirmed PTX‐induced senescence (Figure 4F). Senescent CAFs release a set of proinflammatory factors, growth factors and chemokines termed the senescence‐associated secretory phenotype (SASP) with tumor‐promoting and immunosuppressive functions.^10^ Our study also observed the induction of SASP signature in CAFs (Figure 4G) and tumour biopsies following chemotherapy (Figure 4H). RNA sequencing and Luminex data revealed high expression levels of SASP markers in CAFs after PTX treatment (Figure 4I,J). To further investigate the impact of senescent CAFs on tumour progression, we examined the growth of three‐dimensional spheroids of BC cells and chemotactic migration of immune cells treated with the conditioned medium from CAFs (CAF‐CM) or PTX‐treated CAFs (PTX‐CAF‐CM) (Figure 4K). Compared to the growth of BC cells treated with CAF‐CM, the growth of BC spheroids treated with PTX‐CAF‐CM was significantly increased (Figure 4L). Neutrophil or monocyte attraction was increased by CAF and remained so by PTX treatment of CAF (Figure 4M).

**FIGURE 4:**
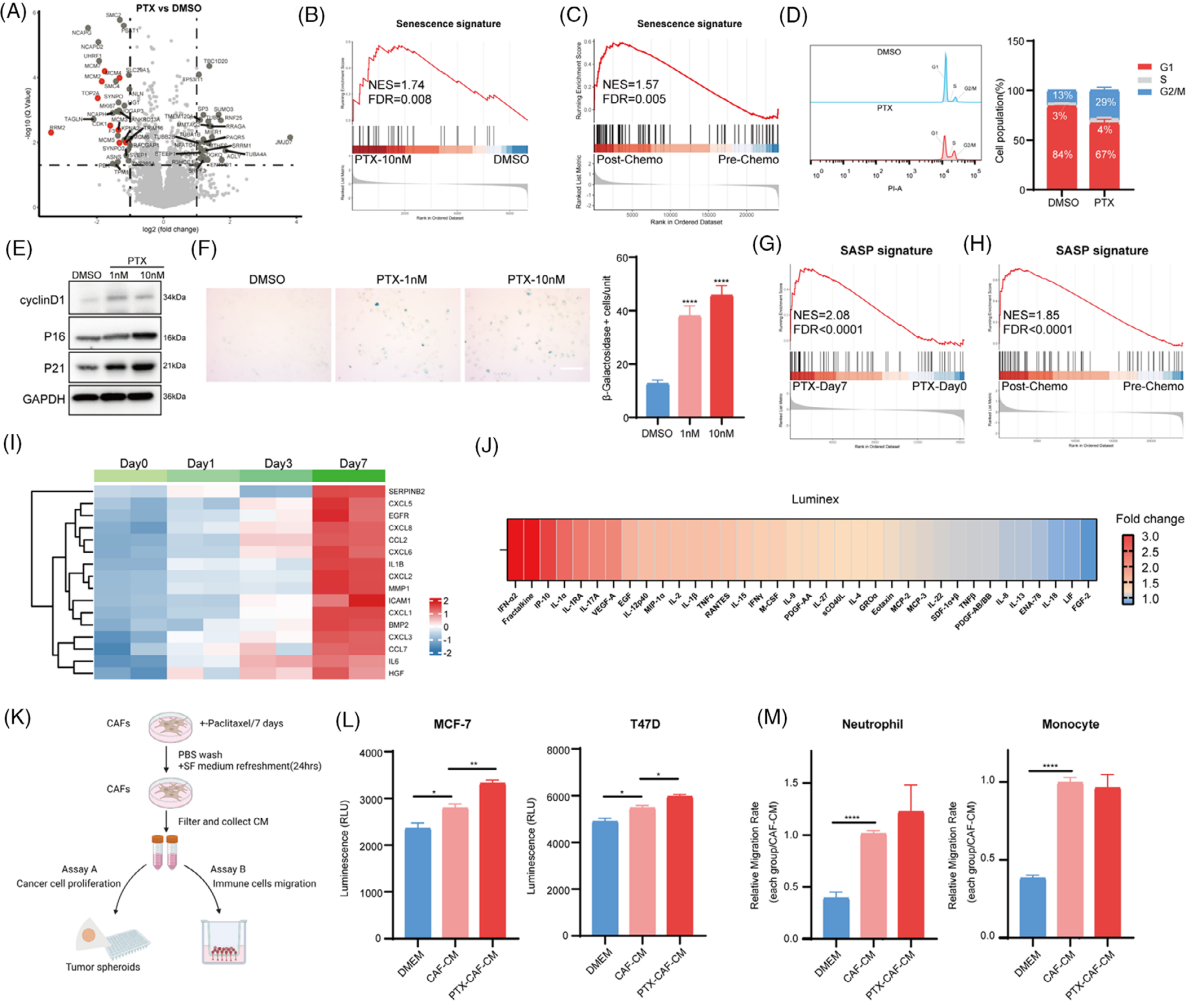
Neoadjuvant chemotherapy (NAC) induces CAF senescence. (A) Volcano plot indicating differentially abundant proteins in cancer‐associated fibroblasts (CAFs) treated with or without paclitaxel (PTX) for 7 days. Cell cycle‐associated proteins are red‐coloured. Differentially abundant proteins were determined based on the absolute value of log2 (Fold change) > 1 and *p*‐value < .05 between the two groups. (B) Gene set enrichment analysis (GSEA) of senescence signature based on the above proteomics data. (C) GSEA of senescence signature among paired post‐chemo versus pre‐chemo tumour biopsies (GSE191127 datasets). (D) Flow cytometry analysis and quantification revealed increased G2/M arrest in breast CAFs treated with PTX for 48 h. (E) Immunoblot analysis of cyclin D1, p16, p21 in CAFs treated with or without PTX for 7 days. GAPDH was used as a loading control. (F) Senescence associated β‐Galactosidase (SA–β‐Gal) staining representative images and quantifications of CAFs treated with or without PTX for 7 days (Scale bar  = 100 µm). (G) GSEA of senescence‐associated secretory phenotype (SASP) signature among CAFs treated with PTX at day 0 and day 7. (H) GSEA of SASP signature among paired post‐chemo versus pre‐chemo tumour biopsies. (I) Heatmap of SASP‐related genes among CAFs treated with PTX at day 0, day 1, day 3 and day 7. (J) Cytokine analysis in the supernatant of CAFs treated with or without PTX for 5 days. The heatmap shows the ranking of the fold change of cytokines between the control group and the PTX‐treated group. (K) Experimental layout of the tumour spheroid growth (assay A) and neutrophils, monocytes transwell migration assay (assay B). (L) Breast cancer cell lines were grown as spheroids and treated with normal medium (DMEM), conditioned media (CM) from CAFs (CAF‐CM) or CM of CAFs pretreated with PTX (PTX‐CAF‐CM). (M) Migration of neutrophils and monocytes towards CAF‐CM, PTX‐CAF‐CM, or DMEM was evaluated. Statistical significance was determined using Student's t‐test (*N* = 3) (each group compared with CAF‐CM group), with * indicating *p*‐value ≤ .05, ** indicating *p*‐value ≤ .01, *** indicating p‐value ≤ .001 and **** indicating *p*‐value ≤ .0001. Significant enrichments were determined based on a normalized enrichment score (NES) threshold of > 1.5 and a false discovery rate (FDR) < 0.05.

In conclusion, our study underscores the critical role of CAFs in impaired NAC response. NAC induces senescence in CAFs, which subsequently promotes tumour progression. Future animal experiments and patient studies should investigate NAC‐induced chemokine secretion and matrix contraction by CAFs and their reciprocal effects in the TME. Targeting CAFs and their senescent state may offer a promising strategy to overcome NAC resistance.

## AUTHOR CONTRIBUTIONS

*Zihan Xia and Olivier De Wever*: contributed to the conception and design of the study. *Zihan Xia*: performed bioinformatic data analysis and wrote the first draft of the article. *Zihan Xia and Stephanie Vermeulen*: performed the experiments. *Ujjwal Suwal, Pekka Rappu, Jyrki Heino and Sandor Dedeyne*: performed proteomics and data analysis. *Stephanie Vermeulen, Felix De Vuyst, An Hendrix, Hannelore Denys and Olivier De Wever*: contributed to revising the manuscript. All authors reviewed the manuscript and approved the submitted version.

## CONFLICT OF INTEREST STATEMENT

The authors declare no conflict of interest.

## FUNDING INFORMATION

This work was supported by the China Scholarship Council (202006160045), Kom Op Tegen Kanker, Stichting Tegen Kanker and Concerted Research Actions of Ghent University.

## ETHICS STATEMENT

The collection of peripheral blood from healthy donors was conducted in accordance with relevant guidelines and approved by the Ethical Committee (EC/2014/0655) of Ghent University Hospital.

## Supporting information

Supporting Information

Supporting Information

## Data Availability

The proteomics data that support the findings of this study have been deposited to the ProteomeXchange Consortium via the PRIDE partner repository at the European Bioinformatics Institute (https://www.ebi.ac.uk/pride/) and are now accessible with the dataset identifier: PXD050005.
