# Supplementary material for: Cancer‐associated fibroblasts mediate resistance to neoadjuvant therapy in breast cancer
Source: Clin Transl Med. 2024 Jul 20;14(7):e1779. doi: 10.1002/ctm2.1779 (PMC11260170; doi:10.1002/ctm2.1779)
Supplement: Supplementary file 1 — Supporting Information [file CTM2-14-e1779-s001.docx]

## Supplementary Table

**Table S1 BC Patients pretreatment characteristics in 4 cohorts**

|  | **JAMA**  GSE25066  **(N=487)** | | **MAQC-II**  GSE20194  **(N=278)** | **NKI**  GSE34138  **(N=177)** | **MD Anderson**  GSE20271  **(N=178)** |
| --- | --- | --- | --- | --- | --- |
| **Age** | | | | | |
| >60 | 88(18%) | 63(23%) | | - | 39(22%) |
| <=60 | 399(82%) | 214(77%) | | - | 139(78%) |
| **Nodal status** | | | | | |
| Positive | 334(69%) | 79(28%) | | - | 59(33%) |
| Negative | 153(31%) | 197(71%) | | - | 118(66%) |
| **T stage** | | | | | |
| 0 | 3(1%) | 3(1%) | | - | 2(1%) |
| 1 | 26(5%) | 23(8%) | | - | 11(6%) |
| 2 | 244(50%) | 147(53%) | | - | 76(43%) |
| 3 | 140(29%) | 50(18%) | | - | 37(21%) |
| 4 | 74(15%) | 52(19%) | | - | 51(29%) |
| **ER status** | | | | | |
| Positive | 285(59%) | 164(59%) | | 114(64%) | 98(55%) |
| Negative | 196(40%) | 114(41%) | | 56(32%) | 80(45%) |
| Indeterminate | 4(1%) | 0 | | 7(4%) | 0 |
| **PR status** | | | | | |
| Positive | 234(48%) | 121(44%) | | 45(25%) | 83(47%) |
| Negative | 246(51%) | 157(56%) | | 85(48%) | 95(53%) |
| Indeterminate | 5(1%) | 0 | | 47(27%) | 0 |
| **Her2 status** | | | | | |
| Positive | 5(1%) | 59(21%) | | 6(3%) | 26(15%) |
| Negative | 465(95%) | 218(78%) | | 171(97%) | 152(85%) |
| Indeterminate | 4(1%) | 1(1%) | | 0 | 0 |
| **Neoadjuvant therapy** | | | | | |
| Sequential T | 91(19%) |  | |  |  |
| Sequential A | 90(18%) |  | |  |  |
| TFAC/TFEC |  | 242(87%) | |  | 91(51%) |
| FAC/FEC |  | 4(1%) | |  | 87(49%) |
| AC |  |  | | 177(100%) |  |
| others | 306(63%) | 32(12%) | |  |  |
| **Chemotherapy Response** | | | | | |
| pCR | 99(20%) | 56(20%) | | 38(21%) | 26(15%) |
| RD | 388(80%) | 222(80%) | | 139(79%) | 152(85%) |

BC, breast cancer. ER, estrogen receptor. PR, progesterone receptor. Her2, human epidermal growth factor receptor 2. Sequential T, sequential taxane–based regimens (then endocrine therapy if ER–positive). Sequential A, sequential anthracycline–based regimens (then endocrine therapy if ER–positive). T, paclitaxel. FAC, 5-fluorouracil, doxorubicin, and cyclophosphamide.  FEC, 5-fluorouracil, epirubicin, and cyclophosphamide. AC, Adriamycin, and cyclophosphamide. pCR, pathological complete response. RD, residual disease.

## Supplementary Figures


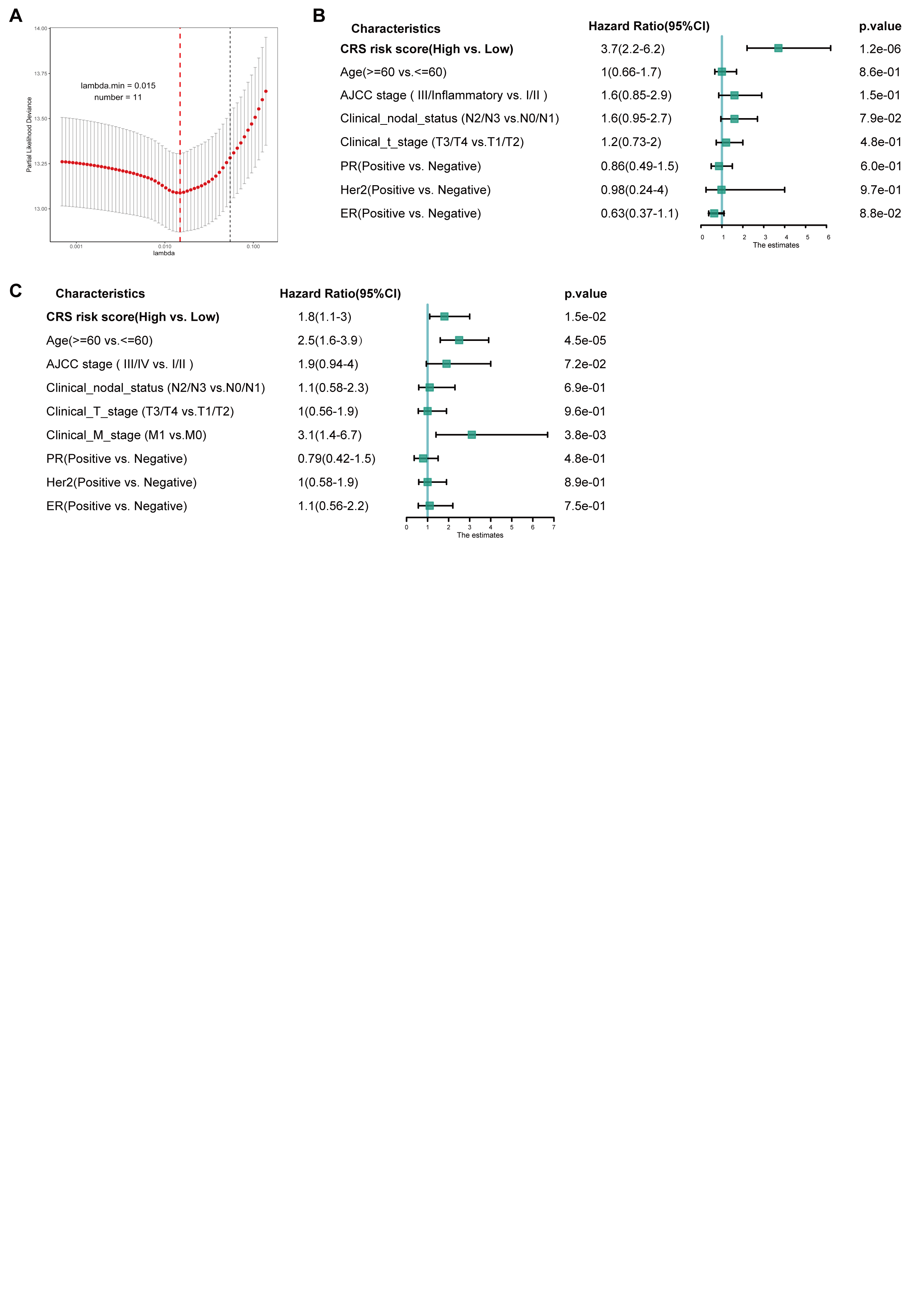


**Figure S1 A.** Screening of 99 genes from the chemo-resistant stroma (CRS) gene set was performed using LASSO regression. **B.** The forest plot illustrates the hazard ratios of the CRS risk score, along with clinicopathologic variables, in the multivariate Cox proportional hazard analyses using the JAMA dataset. **C.** The forest plot displays the hazard ratios of the CRS risk score, as well as clinicopathologic variables, in the multivariate Cox proportional hazard analyses using the TCGA dataset.


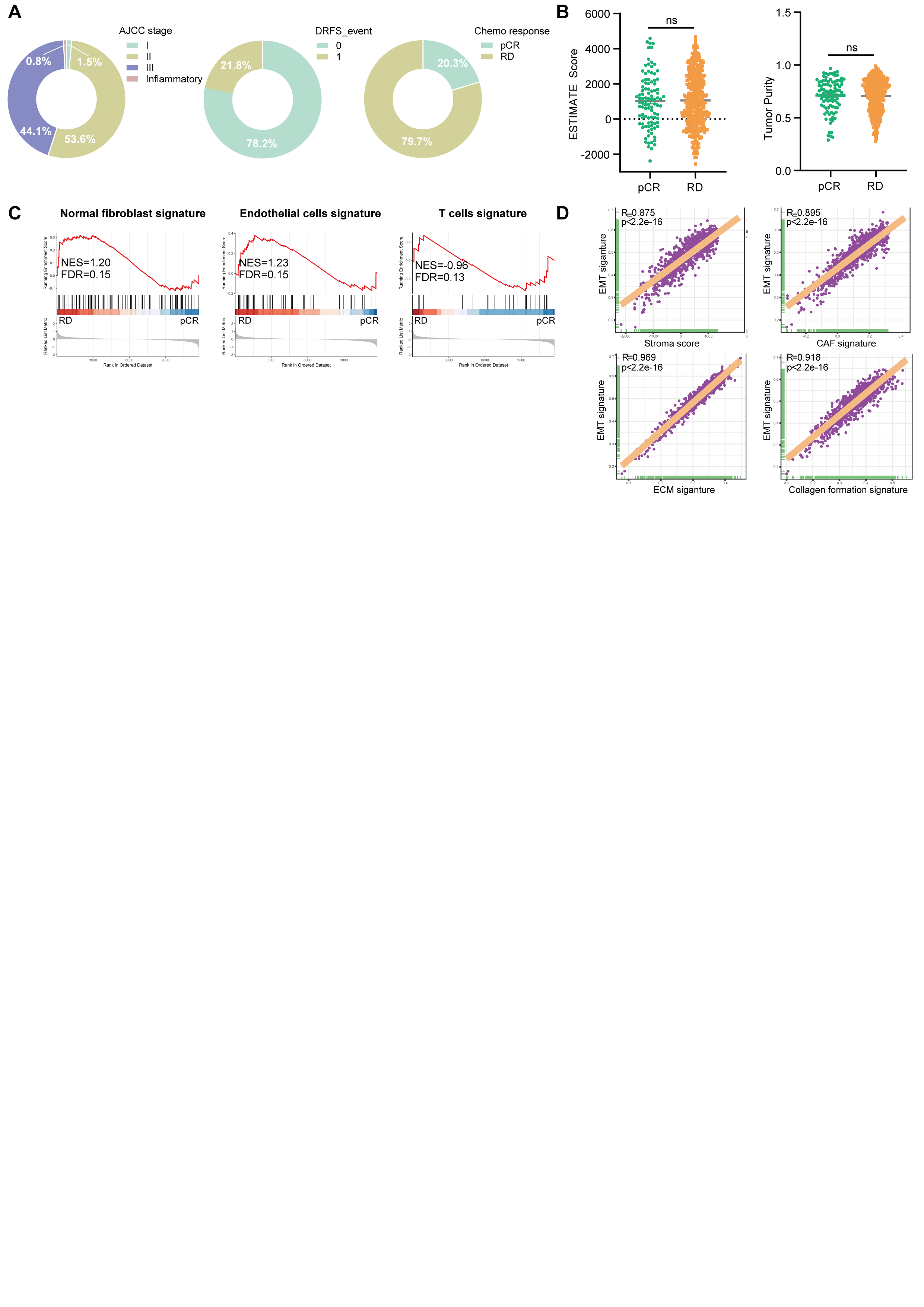


**Figure S2 A.** The pie chart illustrates the distribution of AJCC stage, distant relapse-free survival (DRFS) events, and chemotherapy response in 487 breast cancer patients treated with standard neoadjuvant chemotherapy (JAMA cohort). **B.** The comparison of ESTIMATE score and tumor purity between the pathological complete response (pCR) and residual disease (RD) groups was conducted. Statistical significance was determined using the Wilcoxon test. **C.** Gene set enrichment analysis (GSEA) was performed on transcriptomic data from 487 pre-treatment biopsies (JAMA cohort) to examine the enrichment of normal fibroblast, endothelial cells, and T cells signatures. Significant enrichments were determined based on a normalized enrichment score (NES) threshold of > 1.5 and a false discovery rate (FDR) < 0.05. **D.** Correlation analysis was conducted to assess the relationship between scores for hallmark EMT signatures and scores for stroma signature, CAF signature, ECM signature, and collagen organization signature in TCGA datasets. Purple plotting symbols represent individual samples. Correlation coefficients (R) and p-values were calculated using Spearman's correlation analysis.


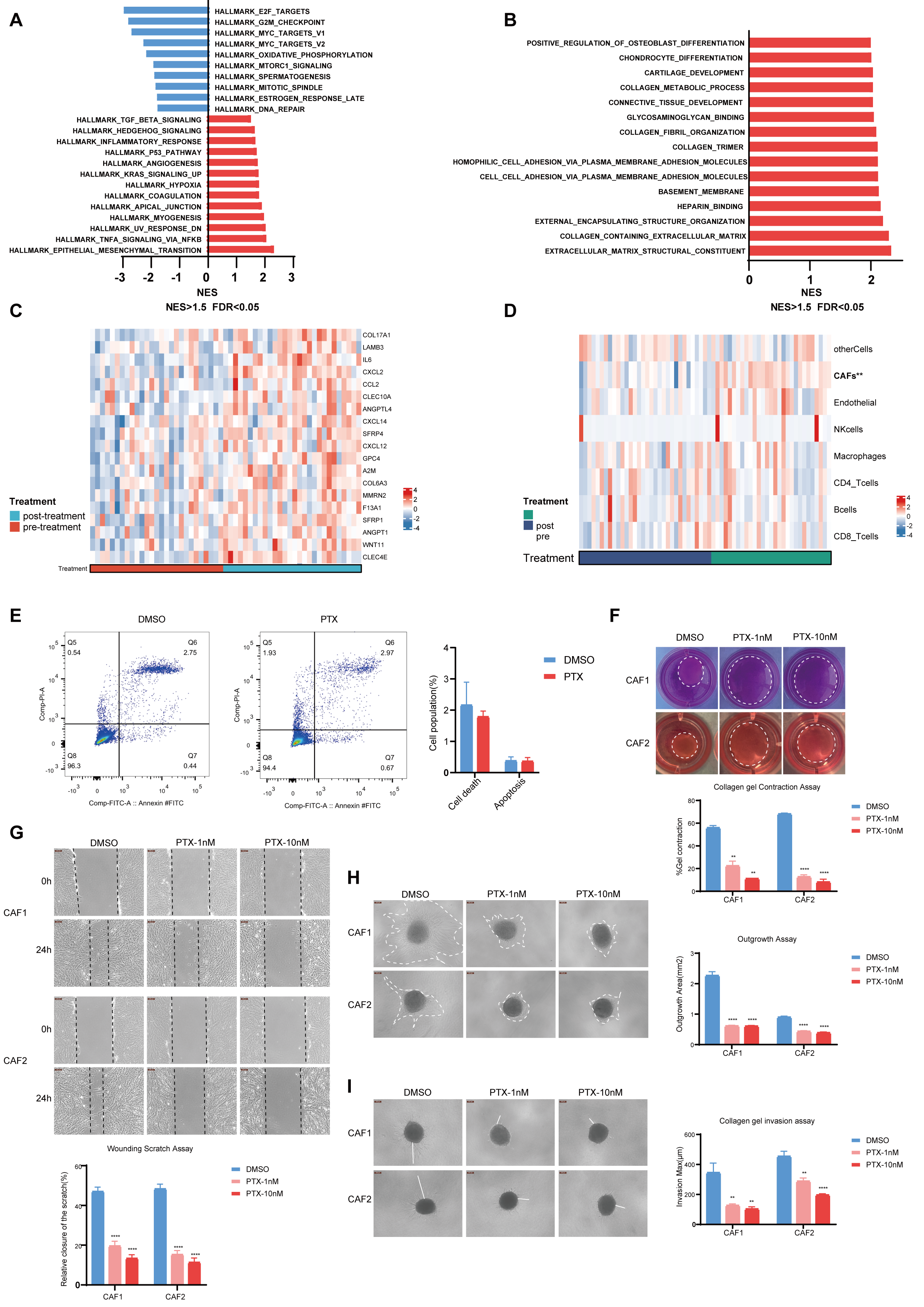
­­­

**FigureS3 A-B.** The bar chart illustrates the significantly enriched hallmark gene sets (A) and significantly enriched gene ontology (GO) signatures (B) in the post-chemotherapy group compared to the pre-chemotherapy group by GSEA using the GSE191127 dataset. Significant enrichments were determined based on an absolute value of normalized enrichment score (NES) >1.5 and a false discovery rate (FDR) <0.05. **C.** Heatmap of differentially extracellular matrix (ECM)-related genes in post-chemo group and pre-chemo group based on GSE114403 cohort. The color of each cell represents the relative abundance of the gene. Differentially ECM -related genes were determined based on log2 (Fold change)>1 and p-value <0.05 between the two groups. **D.** Heatmap of immune cell infiltration using transcriptomic data of 61 biopsies (GSE28844 dataset): 32 pre-chemo samples and 29 post-chemo samples. The analysis was performed using EPIC, and the immune and stromal scores were calculated based on the ESTIMATE algorithm. Differences in immune cell fractions between the two groups were assessed using the Wilcoxon test. (*p-value ≤ 0.05; **p-value ≤ 0.01). **E.** Flow cytometry analysis with Annexin V-PI staining was performed to evaluate the percentage of apoptotic cells and dead cells in breast CAFs treated with 10nM PTX for 48h (DMSO as the control group). **F.** Two breast CAFs were plated into collagen gels with or without PTX (1 nM/10nM) treatment for 24 hours (DMSO as the control group). The area of contracted collagen lattices was quantified as a percentage of the dish surface. **G.** Scratch wound assay was performed on two breast CAFs treated with 1nM/10nM PTX (DMSO as the control group). Images were captured at 0 hours and 48 hours after wounding (Scale bar = 200 μm), and the percentage of wound closure at 48 hours was calculated. **H.** Breast CAFs were grown as 3D spheroids in the ultra-low attachment (ULA) for 48h and then transferred to 96-well plates, with or without PTX (1 nM/10nM) treatment for 3 days (DMSO as the control group). The outgrowth area, including the spheroids, was quantified (Scale bar = 200 μm). **I.** The 3D spheroids of CAFs were transferred from ULA plates to collagen gel with or without PTX (1 nM/10nM) for 3 days (DMSO as the control group). The maximum invasion distance was measured (Scale bar = 200 μm). Representative images and statistical analysis are provided for all functional assays. Each group compared with DMSO group. Statistical significance was determined using Student's t-test (n = 3), with * indicating p-value ≤ 0.05, ** indicating p-value ≤ 0.01, *** indicating p-value ≤ 0.001, and **** indicating p-value ≤ 0.0001.
