# Supplementary material for: Cancer‐associated fibroblasts mediate resistance to neoadjuvant therapy in breast cancer
Source: Clin Transl Med. 2024 Jul 20;14(7):e1779. doi: 10.1002/ctm2.1779 (PMC11260170; doi:10.1002/ctm2.1779)
Supplement: Supplementary file 2 — Supporting Information [file CTM2-14-e1779-s002.docx]

# Supplementary Method

## Experimental Method

**Primary fibroblast isolation**

Tumor fragments (1–3 mm^3^) derived from estrogen receptor-positive breast cancer patients undergoing a mammectomy were collected and seeded in one well of a 6-well tissue coated (TC) plate, precoated with DMEM HG (ThermoFisher, #41965039) medium supplemented with 1% human serum (Merck, #SRP6182), 100 µg/mL choleratoxin (Merck, #C8052), 10^-3^ M hydrocortisone (Merck, #H0888), 2 mg/mL insulin (Merck, #I6634), 1 mg/mL EGF (ThermoFisher, #AF-100-15), 10% fetal bovine serum (FBS) (Pan Biotech, #P30-330), and 100 mg/mL penicillin/streptomycin solution (ThermoFisher, #15070063)^1^. Human fibroblasts attachment and consequent growth ­were observed 5 to 7 days later. Once confluent, fibroblasts were characterized and positive for vimentin (100%) and negative for cytokeratin. They were washed with PBS collected with 0.05% Trypsin EDTA (Life Technologies, #25300062) and seeded into 6-well plates for further experiments. The cells were cultured at 37 °C, 10% CO2, and were split at a ratio of 1:2–1:5 when fully confluent for no more than 6 total passages from the time of isolation.

**Cell culture**

BC cell lines MDA-MB-231, MCF-7 and T47D were grown in DMEM HG medium supplemented with 10% (v/v) FBS, 100 mg/mL penicillin/streptomycin solution and maintained at 37 °C and 5% CO2 in a humidified atmosphere. All cells used were tested monthly using the Mycoalert Mycoplasma Detection Kit (Lonza, #LT07-318) to exclude mycoplasma contamination.

**Spheroid formation**

The single cell suspension containing 1*10^5^ cells/ml of CAFs, or 2*10^4^ cells/ml of cancer cell lines were seeded into U-shaped, 384-well ULA plates (S-bio, # MS-9384UZ), each well containing 80 µl single cell suspension. The 384-well ULA plates were sealed with Breath-Easy semipermeable tape (Diversified Biotech, # BEM-1) to prevent evaporation. The spheroids were cultured at 37 °C in a humidified atmosphere. After 48h of spheroids formation, the treatment was started.

**Collagen contraction assay**

The collagen gel solution was prepared from 3.7 mg/ml Collagen type I from rat tail (Santa Cruz Biotechnology, # sc-136157) and 1:3 mixed with CMF-HBSS in cell culture medium. After that, the pH was adjusted to 7.4 by using 1 M NaOH. All solutions should be kept on ice before use. 2*10^5^ /ml CAFs were added into 500 µl of collagen gel solution and seeded into 24-well plate. Once the gel was solidified (30 minutes at 37°C), it was overlaid with 500 µl medium with or without 1nM/10nM paclitaxel treatment and an equivalent percentage of DMSO as the control group. Gels were photographed after 24h to measure their area with ImageJ software. The percentage of gel contraction was calculated as follows: (well area-gel area)/well area×100%.

**Collagen gel invasion assay**

Collagen gel solution was prepared as described before, 500 µl solution was seeded into each well of a 24-well plate. Once the lower layer of gel was solidified (30 minutes at 37℃), the collagen gel containing the spheroid was seeded on top. The 24-well plate was then placed into an incubator for 30 min to solidify. 500 µl of the medium with or without 1nM/10nM paclitaxel was then added into 24 well plates (an equivalent percentage of DMSO as the control group). After 3 days, spheroids were photographed, and the maximum distance of invasion was analyzed with ImageJ software.

**Scratch wound assay**

CAFs were cultured for 24 h to achieve 100% confluence and a 200 µl sterile pipette tip was used to make a scratch in the cell monolayer. Cells were then washed three times with PBS and incubated in fresh medium with or without 1nM/10nM paclitaxel at 37 °C in a 10% CO2 incubator (an equivalent percentage of DMSO as the control group). The scratch gap area at 48h in each treatment group was measured at four different positions and compared with the gap area at 0 h. The relative closure of the scratch was calculated as follows: (gap area at 0h- gap area at 48h)/ gap area at 0h×100%.

**Outgrowth assay**

48h after seeding, the CAF spheroids were collected and seeded in 96 wells tissue coated (TC) plate. 100 µl of the medium with or without 1nM/10nM paclitaxel was then added into 96-well plate (an equivalent percentage of DMSO as the control group). After 3-5 days, spheroids were photographed, and the outgrow area was analyzed with ImageJ software.

**Flow cytometry**

*Apoptosis.* Annexin A5 Apoptosis Detection Kit was used (BioLegend, #640914) according to the manufacturer’s instructions. Briefly, following 10nM PTX treatment for 48h, the cells were washed and then resuspended in binding buffer at a concentration of 1*10^7^cells/ml. Subsequently, the cells in binding buffer were added to a 5 ml culture tube, followed by gentle vertexing and incubation with 5 µl Annexin V-fluorescein isothiocyanate (FITC) and 10 µl propidium iodide (PI) solution for 15 min at room temperature in the dark. After adding 400 µl binding buffer to each tube, the sample was detected on a BD FACSymphony™ A3 (BD Biosciences) and analyzed using FlowJo software.

*Cell cycle*. Following 10nM PTX treatment for 48h, the cells were collected at a concentration of 1*10^7^ cells/ml and fixed in 70% ethanol at -20 °C overnight. After washing with PBS, cells were incubated with 100 µg/ml Rnase (Merck, #10109142001) and 50 µg/ml PI solution (Merck, #P4170) for 15 min at room temperature in the dark. The sample was then detected on a BD FACSymphony™ A3 (BD Biosciences) and analyzed using FlowJo software.

**ATP assay with Cell Titer-Glo**

For 3D-cultured CAFs and BC cell lines, each spheroid in the culture medium was pipetted into white micro-96-well plates (ThermoFisher, #236108) and an equal volume of CellTiter-Glo® 3.0 Reagent (Promega, #G9683) reagent was added. The 96-well plates were put on an orbital shaker for 5 min. Allow the plate to incubate at room temperature for 10 -20 min to stabilize the luminescent signal. Luminescence readout (Gen5 Data Analysis Software v3.08.01) was performed to record luminescence.

**Luminex**

The supernatant of CAF spheroids treated with vehicle control (DMSO) or 10nM PTX for 5 days was collected and was then passed through a 0.2 µm filter (Novolab, #A37111). The supernatant was processed with the Human Cytokine/Chemokine Array 71-Plex Panel by Eve Technologies. Analysis was performed on a minimum of three biological replicates per condition with subtraction of the growth factor concentrations of medium without cells.

**Western blotting**

CAFs were seeded at 1 *10^6^ cells per ml in 6-well plates overnight. The following day, cells were treated with or without PTX at concentrations of 1nM,10nM, or an equivalent percentage of DMSO as the control group. After 7 days of treatment, cells were washed three times with PBS and 100-200 µl of Laemmli lysis buffer (0.125 M Tris–HCl [pH 6.8], 10% glycerol, 2.3% SDS) were added into each well. Cell lysates were sonicated and centrifuged at 4 °C at 20000 g for 15 min, and the supernatants were transferred to new tubes. Protein concentration of cell lysates was measured using the DC Protein Assay kit (BIO-RAD, #5000112) following the manufacturer's instructions. Cell lysates were mixed with reducing sample buffer (0.5 M Tris-HCl (pH 6.8), 43% glycerol, 9.2% SDS, 5% 2-mercaptoethanol, 5% bromophenol blue) and boiled for 5 min at 95°C. Proteins were separated by SDS–PAGE (SDS polyacrylamide gel electrophoresis) with 25–40 µg of total protein per well, transferred to 0.2-μm-pore nitrocellulose membranes (BIO-RAD,#620112), then blocked in 5%(w/v) milk in PBS and 0.5% Tween-20, and incubated overnight at 4°C with primary antibodies: anti-human p16 INK4A (D7C1M) (1:1000, Rabbit monoclonal, Cell Signaling Technology,#80772), anti-human p21(H-164) (1:100, Rabbit polyclonal, Santa Cruz Biotechnology,#sc-756), Anti-Human Cyclin D1(1:500, mouse monoclonal, Pharmingen,# 554180), anti-human GAPDH (1:2000, mouse monoclonal, Merck Life Science,#G8795). Secondary antibodies: anti-mouse horseradish peroxidase-linked antibody (1:3000, sheep, GE Healthcare Life Sciences, #NA931V), and anti-rabbit horseradish peroxidase-linked antibody (1:8000, donkey, GE Healthcare Life Sciences, #NA934V) were added for 1h at room temperature after 3 times washing with blocking buffer. After 6 times of washing with PBS and 0.5% Tween-20, Blots were detected using the Clarity Western ECL Substrate (BIO-RAD) and visualized on iBright CL 750 (Thermo Fisher Scientific), and images were analyzed using iBright Firmware 1.7.0.

**Proteomics**

*Sample preparation*. CAF spheroids were treated in triplicate by addition of 10 nM PTX or an equivalent percentage of DMSO. After 7 days, the spheroids were collected and washed three times with PBS. Cells were resuspended in lysis buffer (50 mM triethylammonium bicarbonate (TEAB), 5% SDS (pH 7.5)), then sonicated, and clarified with centrifugation of 13,000g for 8 minutes. Each sample was reduced with 20 mM DTT, then warmed the solution first at 95 °C for 10 minutes and then at 60 °C for 1 hour. The reduced proteins were alkylated with 40 mM iodoacetamide (IAA) in the dark for 30 minutes. Then samples were acidified with 1.2% aqueous phosphoric acid and mixed with 6× volumes of 90% methanol, 100 mM TEAB, pH 7.1 (S-Trap binding buffer). The samples were loaded onto the filter of the S-Trap micro column (ProtiFi) and centrifuged at 4000×g for 30 seconds. The flow-through was discarded each time and the samples were washed 9 times with the S-Trap binding buffer. Proteins trapped in the S-Trap column were digested with Trypsin/Lys-C Mix (Promega, USA) at 47°C for 1 hour at a protein-to-enzyme ratio of 1:25 (w/w). The digested peptides were eluted with buffers in order: 50 mM TEAB pH 8.0, 0.2% aqueous formic acid, and 50% acetonitrile/0.2% aqueous formic acid. Then the samples were lyophilized using a SpeedVac (Thermo Fisher Scientific) with cold trap and desalted with C18 tips (Empore). Finally, the desalted peptides were again SpeedVac dried and dissolved in 0.1% formic acid for liquid chromatography-mass spectrometry analysis.

*Liquid chromatography-tandem mass spectrometry.* The peptide samples were analyzed utilizing a nanoflow HPLC system (Easy-nLC1200, Thermo Fisher Scientific) coupled to an Orbitrap Fusion Lumos mass spectrometer (Thermo Fisher Scientific, Bremen, Germany). This setup incorporated a high-field asymmetric waveform ion mobility spectrometry (FAIMS) device positioned between the nano-electrospray ionization source and the mass spectrometer. Compensation voltages of -50 V and -70 V were employed. Peptides underwent initial loading onto a trapping column, followed by inline separation on a 15 cm C18 column (75 μm × 15 cm, ReproSil-Pur 3 μm 120 Å C18-AQ, Dr. Maisch HPLC GmbH, Ammerbuch-Entringen, Germany). Separation was achieved using a 120-minute, 2-step gradient consisting of solvents A (0.1% formic acid) and B (acetonitrile/water (95:5(v/v)) with 0.1% formic acid). The gradient profile involved: 5–21% B in 62 minutes, 21-36% B in 48 minutes, 36-100% in 5 minutes, followed by a 5-minute hold at 100% B. Data-independent acquisition (DIA) guided analysis, with each cycle comprised of a full scan (395-1000 m/z, 120,000 resolution, 50 ms maximum injection time, 7e5 AGC target). All DIA scans utilized DIA MS2 scans (30,000 resolution, 1e6 AGC target, 52 ms maximum injection time), divided into 30 variable window schemes with 1 m/z overlap. DIA spectra were obtained at both -50 V and -70 V FAIMS compensation voltages. To generate a chromatogram library, a pool of all experimental samples was constructed. This pool served for six gas phase fractionated DIA runs, employing identical LC and DIA settings but with an overlapping isolation window of 4 m/z and a mass range of 100 m/z (400-500 m/z, 500-600 m/z, 600-700 m/z, 700-800 m/z, 800-900 m/z, and 900-1000 m/z).

**SA–β-Galactosidase staining assay**

Senescence β-Galactosidase Staining Kit (Cell Signaling Technology, #9860) was used following the manufacturer’s protocol. Briefly, following 10nM PTX treatment for 7 days, CAFs were fixed with the fixative solution and stained with a staining solution mix (Staining Solution, solution A, solution B, 20 mg/ml X-gal in DMSO) overnight at 37 °C. Cells were then washed twice with PBS, and the percentage of stained cells was determined.

**CAF conditioned media**

For conditioned media experiments, CAFs were treated with or without 10 nM PTX in DMEM containing 10% FBS (an equivalent percentage of DMSO as the control group). After 72h treatment, the medium was then removed and replaced with serum free medium for another 48h. CAF-conditioned medium (CM) was then collected, centrifuged to remove debris, and filtered through a 0.2 µm filter.

**Neutrophil/Monocyte Isolation**

Human neutrophils and monocytes were isolated from the peripheral blood of healthy donors using Lymphocyte®-poly (Tebubio, #CL5071) density gradient centrifugation^2^. After centrifugation, the monocyte at the upper band and the neutrophils at the lower band were resuspended in DMEM supplemented with 10% FBS and cultured in 24-well plate at 37 °C and 5% CO2.

**Co-culture assay**

*Neutrophil/Monocyte Migration****.***1*10^5^/ml isolated neutrophils and 5*10^4^/ml monocytes are seeded in the upper chamber of IncuCyte® Clearview 96-well Cell Migration Plates (Sartorius, #4599) in serum-free medium. The chemoattractant of conditioned medium from CAF with or without 10nM PTX treatment are added to the lower chamber. After 4h, immune cell migration into the lower chamber is quantified by total cellular ATP levels via a luminescent-based method (Cell Titer-Glo 2D).

*Cancer cell proliferation.* Once cancer cell spheroids formed, 40 µl supernatant of each well was removed and then 40 µl of conditioned media of CAFs (1:1 v/v) was added. After 5 days of co-culture, total cellular ATP levels were quantified via a luminescent-based method (Cell Titer-Glo 3D).

## Bioinformatic method

**Dataset collection and preprocessing**

From The Cancer Genome Atlas (TCGA) database (<https://cancergenome.nih.gov/>), RNA sequencing data of 1095 BRCA patients with clinicopathologic characteristics were downloaded for subsequent bioinformatic analysis. The count data were then converted to log2 (TPM+1) data. To identify possible predictive markers that may be responsible for NAC response, gene expression data and the matched clinical information of BC patients before NAC from JAMA (GSE25066)^3^, MAQC-II (GSE20194)^4^, MD Anderson (GSE20271)^5^, NKI (GSE34138)^6^ datasets were collected from the Gene Expression Omnibus (GEO) (<https://www.ncbi.nlm.nih.gov/geo/>). Patient demographics and clinicopathologic characteristics for all patient cohorts mentioned above are shown in Supplementary **Table S1**. To explore the mechanism through which NAC regulates TME, transcriptomic data of BC patients before and after NAC from GSE28583^7^, GSE191127^8^, and GSE114403^9^ datasets were collected from the GEO. To explore whether NAC induce senescence in CAFs, transcriptomic data of CAFs treated with PTX at day 0, day 1, day 3, and day 7 from GSE23399^10^ datasets were also collected from the GEO. Data were analyzed with R (version 4.2.2) and R Bioconductor packages. Normalization and differential expression analysis for RNA-sequencing data were performed using the DESeq2 R package, for Microarray data were performed using the limma R package.

**Estimation of Stroma score and Immune cell infiltration**

We estimated stroma cell infiltration by employing several algorithms in R: each sample’s ESTIMATE score, immune score, stromal score and tumor purity were determined using the R package ESTIMATE^11^. The deconvolution-based algorithm was utilized to quantify the relative abundance of each stromal cell infiltration, and the EPIC package was employed to determine the infiltration of 8 different stromal cell types^12^.

**Survival analysis**

Kaplan-Meier survival analyses were performed with survival and survminer R package. The cut-off value of continuous variables in the survival data was split by median. p-values were calculated by log-rank test.

**Gene set enrichment analysis**

Gene set enrichment analysis (GSEA) was performed using the org.Hs.eg.db and clusterProfiler packages. The following gene sets were downloaded from the MSigDB database (version 7.1, Broad Institute, <https://www.gsea-msigdb.org>) and analyzed: 50 Hallmarks gene sets, Reactome extracellular matrix organization, Reactome_collagen formation, GO_extracellular matrix, Reactome_DNA damage telomere stress-induced senescence (used for Figure 4B), Fridman_senescence up (used for Figure 4C). CAF, normal fibroblasts, endothelial cells, T cells, and SASP genesets were collected from the literature and analyzed^13,14^. The chemo-resistant stroma gene set was defined based on differentially expressed genes in laser capture micro-dissected stroma cells from RD patients (GSE143846^15^ dataset, log2(Foldchange)>1 and p-value < 0.05). Significant enrichments were determined based on an absolute value of normalized enrichment score (NES) threshold >1.5 and a false discovery rate (FDR) <0.05.

**Prognostic model**

99 genes from the chemo-resistant stroma (CRS) gene set were screened out by univariate Cox analysis (P<0.05) and the Lasso Cox regression model to obtain 11 potential CRS-based genes^16^. Subsequently, we created a risk model using the regression coefficients from the multivariate Cox regression analysis in the GSE25066 dataset as our discovery cohorts and then validated in the TCGA dataset. The formula for the risk model was established as follows: Risk score = H0t∗exp[expression (gene [1]) ×coefficients(gene[1])+expression(gene[2]) × coefficient(gene[2])+ … +expression(gene[11])× coefficient (gene[11])]. Kaplan‒Meier survival analysis and multivariate Cox analysis were performed to evaluate the predictive value of the risk model.

**Statistical analysis**

Experimental data analyses were performed using GraphPad Prism 9 software. Bioinformatic data were performed using R (version 4.2.2). The unpaired student’s t-test and chi-square test were used to compare continuous and categorical variables. The Wilcoxon test was used to compare signature scores or immune cell infiltration between groups. In addition, Spearman analysis was performed to assess the correlation between different variables. A p-value of< 0.05 was considered statistically significant.

## Reference

1. Primac I, Maquoi E, Blacher S, et al. Stromal integrin α11 regulates PDGFRβ signaling and promotes breast cancer progression. *J Clin Invest*. 2019;129(11):4609-4628. doi:10.1172/JCI125890

2. Cui C, Schoenfelt KQ, Becker KM, Becker L. Isolation of polymorphonuclear neutrophils and monocytes from a single sample of human peripheral blood. *STAR Protoc*. 2021;2(4):100845. doi:10.1016/j.xpro.2021.100845

3. Hatzis C, Pusztai L, Valero V, et al. A genomic predictor of response and survival following taxane-anthracycline chemotherapy for invasive breast cancer. *JAMA*. 2011;305(18):1873-1881. doi:10.1001/jama.2011.593

4. Popovici V, Chen W, Gallas BG, et al. Effect of training-sample size and classification difficulty on the accuracy of genomic predictors. *Breast Cancer Res BCR*. 2010;12(1):R5. doi:10.1186/bcr2468

5. Tabchy A, Valero V, Vidaurre T, et al. Evaluation of a 30-gene paclitaxel, fluorouracil, doxorubicin, and cyclophosphamide chemotherapy response predictor in a multicenter randomized trial in breast cancer. *Clin Cancer Res Off J Am Assoc Cancer Res*. 2010;16(21):5351-5361. doi:10.1158/1078-0432.CCR-10-1265

6. de Ronde JJ, Lips EH, Mulder L, et al. SERPINA6, BEX1, AGTR1, SLC26A3, and LAPTM4B are markers of resistance to neoadjuvant chemotherapy in HER2-negative breast cancer. *Breast Cancer Res Treat*. 2013;137(1):213-223. doi:10.1007/s10549-012-2340-x

7. Vera-Ramirez L, Sanchez-Rovira P, Ramirez-Tortosa CL, Quiles JL, Ramirez-Tortosa M, Lorente JA. Transcriptional shift identifies a set of genes driving breast cancer chemoresistance. *PloS One*. 2013;8(1):e53983. doi:10.1371/journal.pone.0053983

8. Hoogstraat M, Lips EH, Mayayo-Peralta I, et al. Comprehensive characterization of pre- and post-treatment samples of breast cancer reveal potential mechanisms of chemotherapy resistance. *NPJ Breast Cancer*. 2022;8(1):60. doi:10.1038/s41523-022-00428-8

9. Li X, Warren S, Pelekanou V, et al. Immune profiling of pre- and post-treatment breast cancer tissues from the SWOG S0800 neoadjuvant trial. *J Immunother Cancer*. 2019;7(1):88. doi:10.1186/s40425-019-0563-7

10. Chan TS, Hsu CC, Pai VC, et al. Metronomic chemotherapy prevents therapy-induced stromal activation and induction of tumor-initiating cells. *J Exp Med*. 2016;213(13):2967-2988. doi:10.1084/jem.20151665

11. Yoshihara K, Shahmoradgoli M, Martínez E, et al. Inferring tumour purity and stromal and immune cell admixture from expression data. *Nat Commun*. 2013;4(1):2612. doi:10.1038/ncomms3612

12. Racle J, Gfeller D. EPIC: A Tool to Estimate the Proportions of Different Cell Types from Bulk Gene Expression Data. *Methods Mol Biol Clifton NJ*. 2020;2120:233-248. doi:10.1007/978-1-0716-0327-7_17

13. Geldhof V, de Rooij LPMH, Sokol L, et al. Single cell atlas identifies lipid-processing and immunomodulatory endothelial cells in healthy and malignant breast. *Nat Commun*. 2022;13(1):5511. doi:10.1038/s41467-022-33052-y

14. Linares J, Sallent-Aragay A, Badia-Ramentol J, et al. Long-term platinum-based drug accumulation in cancer-associated fibroblasts promotes colorectal cancer progression and resistance to therapy. *Nat Commun*. 2023;14(1):746. doi:10.1038/s41467-023-36334-1

15. Katayama MLH, Vieira RA da C, Andrade VP, et al. Stromal Cell Signature Associated with Response to Neoadjuvant Chemotherapy in Locally Advanced Breast Cancer. *Cells*. 2019;8(12):1566. doi:10.3390/cells8121566

16. Tibshirani R. The lasso method for variable selection in the Cox model. *Stat Med*. 1997;16(4):385-395. doi:10.1002/(sici)1097-0258(19970228)16:4<385::aid-sim380>3.0.co;2-3
